# Abnormal biochemical indicators of neonatal inherited metabolic disease in carriers

**DOI:** 10.1186/s13023-024-03138-5

**Published:** 2024-04-04

**Authors:** Fang Guo, Lingna Zhou, Feng Zhang, Bin Yu, Yuqi Yang, Zhiwei Liu

**Affiliations:** Changzhou Maternal and Child Health Care Hospital, Changzhou Medical Center, Nanjing Medical University, No.16 Ding Xiang Road, Changzhou, Jiangsu Province China

**Keywords:** Neonatal inherited metabolic disease, Gene variant carriers, Biochemical indicators

## Abstract

**Background:**

Traditional biochemical screening for neonatal inherited metabolic diseases has high false-positive rates and low positive predictive values, which are not conducive to early diagnosis and increase parents’ anxiety. This study analysed the relationship between gene variant carriers and their biochemical indicators in traditional biochemical screening, aiming to find explanations for false positives in newborns.

**Results:**

This retrospective study included 962 newborns. Newborns underwent traditional biochemical screening at birth using blood staining and genomic sequencing of their stored blood staining using the NeoSeq Pro panel, which was able to detect 154 pathogenic genes and 86 diseases. A total of 632 newborns were carriers of gene variants. 56% of congenital hypothyroidism carriers had higher thyroid-stimulating hormone levels than normal newborns. Abnormal biochemical indices were detected in 71% of carriers of organic acid metabolic diseases, 69% of carriers of amino acid metabolic diseases, and 85% of carriers of fatty acid β oxidation disorders. In carriers associated with organic acid metabolic diseases, the propionylcarnitine (C3), C3/acetylcarnitine (C2), and methylmalonylcarnitine (C4DC) + 3-hydroxyisovalerylcarnitine (C5OH) levels were higher than those in non-carriers (C3: 4.12 vs. 1.66 µmol/L; C3/C2: 0.15 vs. 0.09; C4DC + C5OH: 0.22 vs. 0.19 µmol/L). In carriers associated with amino acid metabolic diseases, phenylalanine levels were higher than those in non-carriers (68.00 vs. 52.05 µmol/L). For carriers of fatty acid β oxidation disorders, butyrylcarnitine levels were higher than those in non-carriers (0.31 vs. 0.21 µmol/L), while the free carnitine levels were lower than those in non-carriers (14.65 vs. 21.87 µmol/L). There was a higher occurrence of carriers among newborns who received false-positive results for amino acid metabolic diseases compared to those who received negative results (15.52% vs. 6.71%). Similarly, there was a higher occurrence of carriers among newborns who received false-positive results for fatty acid β oxidation disorders compared to those who received negative results (28.30% vs. 7.29%).

**Conclusions:**

This study showed that the carriers comprised a large number of newborns. Carriers had abnormal biochemical indicators compared with non-carriers, which could explain the false-positive rate for newborns using traditional newborn biochemical screening, especially in amino acid metabolic and fatty acid β oxidation disorders.

**Supplementary Information:**

The online version contains supplementary material available at 10.1186/s13023-024-03138-5.

## Background

Newborn screening (NBS) is a successful public health program used for early disease detection, diagnosis, and intervention. Screening neonates for inherited metabolic diseases (IMDs) is an important part of NBS [1]. IMD refers to a group of diseases with defects in the functions of enzymes, receptors, and carriers caused by gene variants that lead to abnormalities in the synthesis, metabolism, transport, and storage of biochemical substances, resulting in a series of clinical symptoms [2]. There are a wide variety of IMDs that, if not treated promptly, can cause mental and physical developmental abnormalities and even lead to the death of newborns. The first neonatal IMD screened for was phenylketonuria in 1959 [3], and NBS was dominated by single disease screening until the application of tandem mass spectrometry (MS/MS) technology expanded IMDs to more than 40 different diseases [4]. MS/MS is a high throughput technology with high sensitivity and specificity, which plays an important role in the early diagnosis of amino acid, organic acid metabolic, and fatty acid β oxidation disorders.

Most studies have reported that MS/MS detection methods have high false-positive rates and low positive predictive values because of their high sensitivity [5]. Therefore, it is necessary to develop improved NBS methods that can reduce false-positive rates or find other explanations for false positives to decrease anxiety and stress in parents of newborns. In recent years, with the rapid development of next-generation sequencing (NGS) [6], genetic testing has played an important role in newborn disease detection, which not only expands the types of diseases that can be screened but also aids in the diagnosis of neonatal IMDs. In our previous study, we found that 30% of false-positive newborns identified using traditional biochemical screening methods were carriers of gene variants [7]. However, due to the small number of cases, there has been no detailed analysis of the relationship between gene variant carriers and biochemical indices. Therefore, we expanded the sample size in this study and investigated whether the carried gene variants could explain the high false-positive rates in newborns.

## Results

**Fig. 1:**
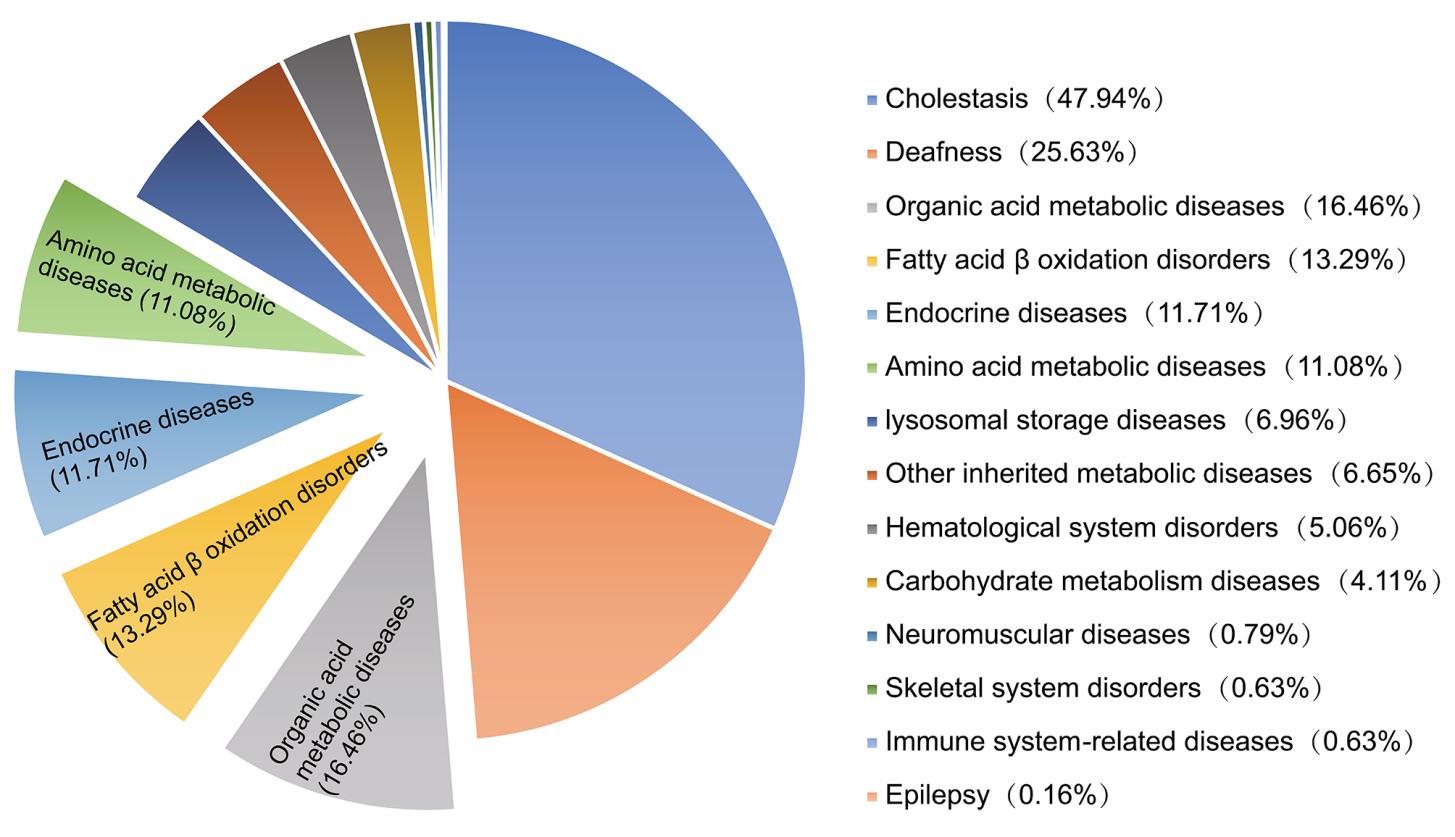
Classification of diseases associated with 632 gene variant carriers Legend Variants were grouped into 14 categories to encompass low-frequency gene variants and a variety of diseases

Of the 962 newborn infants analysed, 68 were diagnosed with positive gene results using NGS and were excluded from subsequent analyses. A total of 632 (65.7%) newborns carrying gene variants were defined as the carrier group, and 262 newborns that did not carry variants were defined as the non-carrier group. In the carrier group, 976 variants were identified, of which 465 (47.6%) were pathogenic, 471 (48.3%) were likely pathogenic, and 40 (4.1%) were variants of uncertain significance. A total of 383 (60.6%) infants carried single variants, and 249 (39.4%) infants carried two or more variants. These variants included 94 genes; the top ten high-frequency gene variants in the carriers are shown in Table 1. Among them, variants of *DUOX2*, *SLC22A5*, *PAH*, and *ACADS* are associated with diseases in traditional newborn biochemical screening and are ranked third, fourth, seventh, and ninth, respectively. Because the carrying rate of many variants was extremely low and a variety of diseases were involved, we grouped these diseases into 14 categories (Fig. 1 and Supplementary Table 1). Cholestasis and deafness had the highest carrying rates, accounting for 47.94% and 25.63%, respectively. The carrying rate of neonatal genetic metabolic diseases, including organic acid metabolic diseases, fatty acid β oxidation disorders, endocrine diseases (mainly congenital hypothyroidism (CH)), and amino acid metabolic diseases, was relatively high, accounting for 16.46%, 13.29%, 11.71%, and 11.08%, respectively.

**Table 1:** Top ten high-frequency gene variants of carriers and the related diseases

	Top ten high-frequency gene variants	Related diseases	NO. of case(percentage)
1	UGT1A1	Crigler-najjar syndrome	312 (49.4)
2	GJB2	Deafness	132 (20.9)
3	DUOX2	Congenital Hypothyroidism	64 (10.1)
4	SLC22A5	Primary carnitine deficiency	31 (4.9)
5	GALC	Krabbe disease	29 (4.6)
6	ATP7B	Wilson disease	27 (4.3)
7	PAH	Hyperphenylalaninemia	22 (3.5)
8	HBA1, HBA2	Alpha-thalassemia	21 (3.3)
9	ACADS	Short-chain acyl-CoA dehydrogenase deficiency	16 (2.5)
10	SLC26A4	Deafness	16 (2.5)

We analysed the differences in biochemical indicators between the carriers and normal newborns. The median of the biochemical indicators in normal newborns was calculated using 192,110 cases with negative results in traditional newborn biochemical screening in Changzhou from 2017 to 2022. As shown in Table 2, in 73 cases of CH gene variants, 56% of carriers had higher levels of thyroid-stimulating hormone (TSH) than normal newborns. In 103 cases of organic acid metabolic disease carriers, 71% of carriers had higher biochemical indicators than normal newborns, and the most common variant was associated with methylmalonic acidemia/propionic acidemia. Among the 72 carriers of amino acid metabolic disease gene variants, 69% had biochemical indicators higher or lower than the normal newborns, and the most common variant was associated with hyperphenylalaninemia. Of the 84 carriers of fatty acid β oxidation disorder gene variants, 85% had biochemical indicators higher or lower than the normal newborns, and the most common variant was associated with primary carnitine deficiency.

**Table 2:** Relationship between biochemical indicators for neonatal inherited metabolic diseases and gene variants

	Case of carriers	Related disease	Main abnormal biochemical indicators	above or below (^a^) median of the normal newborns
Endocrine diseases	73	Congenital hypothyroidism	TSH	**41(56%)**
Organic acid metabolic disease	58	Methylmalonic acidemia, Propionicacidemia	C3;C3/C2	48;46
	5	Glutaric acidemia I	C5DC + C6OH	3
	26	Holocarboxylase synthetase deficiency, Biotinidase deficiency, 3-Hydroxy-3-methylglutaryl-CoA synthase-2 deficiency, 3-Methylcrotonyl-CoA carboxylase deficiency	C4DC + C5OH	15
	1	MalonyI-CoA decarboxylase deficiency	C3DC + C4OH	0
	13	Isovaleric academia, 2-Methylbutyryl-CoA dehydrogenase deficiency, Isobutyryl-CoA dehydrogenase deficiency	C5	7
Total	103			**73(71%)**
Amino acid metabolic disease	3	Maple syrup urine disease	Leu + Ile + Pro-OH	1
	2	Tyrosinemia	Tyr	2
	31	Hyperphenylalaninemia	Phe	24
	10	Homocystinuria, Hypermethioninemia	Met	6
	3	Nonketotic hyperglycinemia	Gly	1
	19	Argininosuccinic aciduria, Citrullinemia Type I, Citrin deficiency	Cit	13
	2	Hyperornithinemia-hyperammonemia-homocitrullinemia syndrome	Orn	1
	2	Carbamoyl phosphate syntetase I deficiency	Cit	2^a^
Total	72			**50(69%**)
Fatty acid β oxidation disorder	8	Very long-chain acyl-CoA dehydrogenase deficiency	C14:1	8
	3	Long-chain 3-hydroxyacyl-CoA dehydrogenase deficiency, Mitochondrial trifunctional protein deficiency	C16OH	3
	5	Medium chain acyl-CoA dehydrogenase deficiency	C8	4
	16	Short-chain acyl-CoA dehydrogenase deficiency	C4	14
	9	Glutaric acidemia II	C5, C4	5;7
	2	β-ketothiolase deficiency	C5:1;C4DC + C5OH	0;2
	7	Carnitine palmitoy ltransferase II deficiency, Carnitine-acylcarnitine translocase deficiency	C16;C18	4;4
	34	Primary carnitine deficiency	C0	29^a^
Total	84			**71(85%)**

**Fig. 2:**
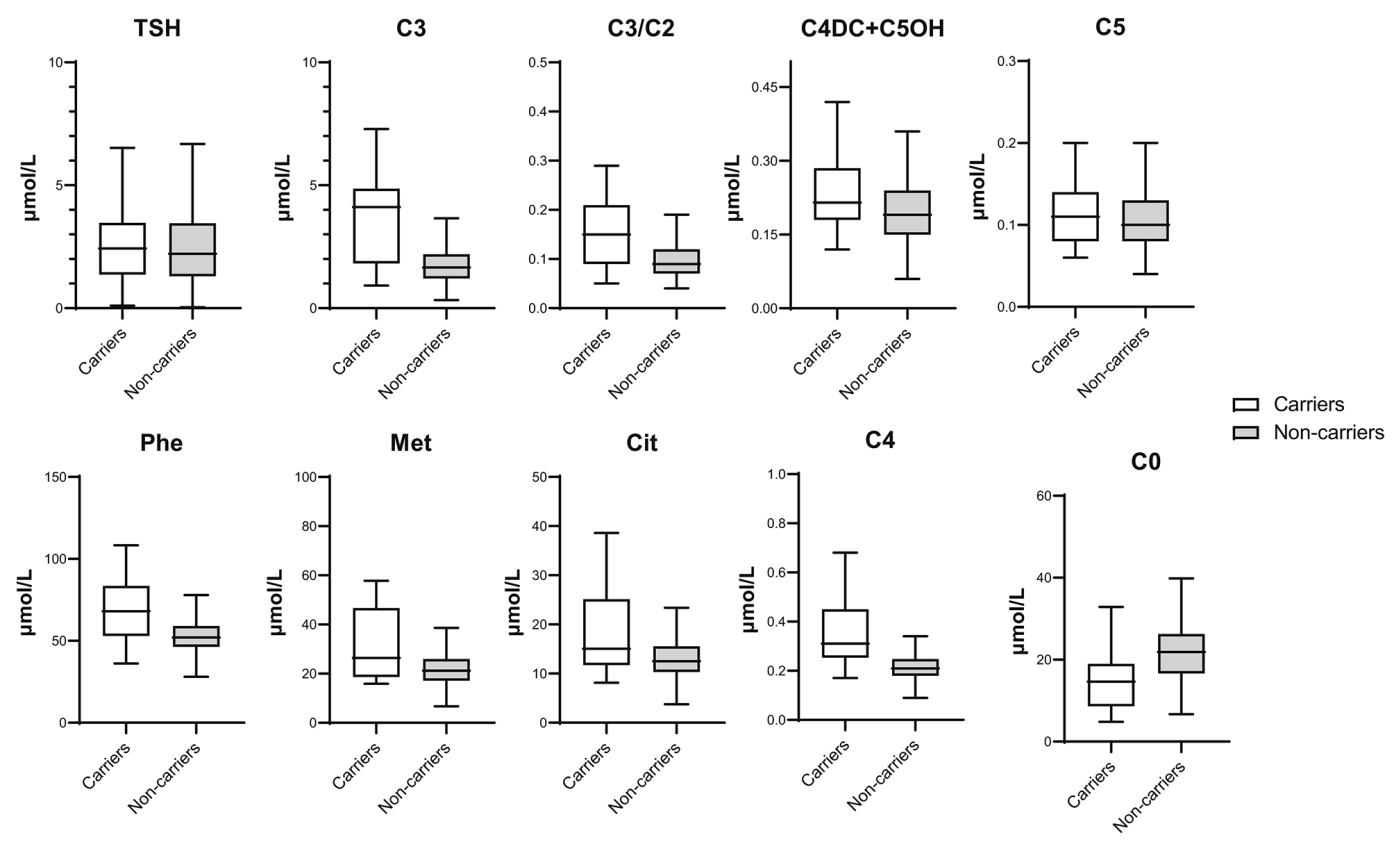
Differences in biochemical indicators between the carrier and non-carrier groups Legend TSH, thyroid-stimulating hormone; C3, propionylcarnitine; C2, acetylcarnitine; C4DC, acetylcarnitine; C5OH, 3-hydroxyisovalerylcarnitine; Phe, phenylalanine; Met, methionine; Cit, citrullinaemia; C0, free carnitine

We selected diseases with relatively high carrying rates and analysed the differences in biochemical indicators among carriers, non-carriers, and the normal population. First, we compared the differences between carrier and non-carrier groups. In carriers of organic acid metabolic diseases, levels of propionylcarnitine (C3), C3/acetylcarnitine (C2), and methylmalonylcarnitine (C4DC) + 3-hydroxyisovalerylcarnitine (C5OH) were higher than those in non-carriers (C3: 4.12 vs. 1.66 µmol/L, *p* < 0.001; C3/C2: 0.15 vs. 0.09, *p* < 0.001; C4DC + C5OH: 0.22 vs. 0.19 µmol/L, *p* = 0.032) (Table 3; Fig. 2). In carriers of amino acid metabolic diseases, levels of phenylalanine (Phe) were higher than those in non-carriers (68.00 vs. 52.05 µmol/L, *p* = 0.001); methionine (Met; *p* = 0.097) and citrullinaemia (Cit; *p* = 0.077) also showed the same trends. For carriers of fatty acid β oxidation disorders, levels of butyrylcarnitine (C4) were higher than those in non-carriers (0.31 vs. 0.21 µmol/L, *p* = 0.008), while free carnitine (C0) levels showed the opposite result (14.65 vs. 21.87 µmol/L, *p* < 0.001).

Due to the large sample size of the normal population and the small sample size of carriers and non-carriers, a direct comparison of statistical differences among the three groups would reduce the statistical power. Therefore, we used the median of the normal population and calculated the ratio of carriers and non-carriers to the median to compare these groups with the normal population. In organic acid metabolic diseases, the C3 and C3/C2 levels of carriers were 2.29 and 1.68 times higher than those of the normal population, respectively. In fatty acid β oxidation disorders, the C4 levels of carriers were 1.8 times higher than those of the normal population, while the C0 levels of carriers were 0.69 times lower than those of the normal population. Almost all biochemical indicators in the non-carrier group were similar to those in the normal population.

**Table 3:** Differences in biochemical indicators between the carriers, non-carriers, and the normal population

	Related disease	Case of carriers	Main abnormal biochemical index	Carrier group	Non-carrier group	the ratio of carriers to the normal newborns	the ratio of non-carriers to the normal newborns	*P*
Endocrine disease	Congenital hypothyroidism	73	TSH	2.43(1.37,3.47)	2.21(1.30,3.46)	1.19 ± 0.79	1.09 ± 0.69	0.290
Organic acid metabolic disease	Methylmalonic academia, Propionicacidemia	58	C3	4.12(1.82,4.86)	1.66(1.20,2.20)	2.29 ± 1.05	1.17 ± 0.64	< 0.001
			C3/C2	0.15(0.09,0.21)	0.09(0.07,0.12)	1.68 ± 0.75	1.12 ± 0.41	< 0.001
	Holocarboxylase synthetase deficiency, Biotinidase deficiency, 3-Hydroxy-3-methylglutaryl-CoA synthase-2 deficiency, 3-Methylcrotonyl-CoA carboxylase deficiency	26	C4DC + C5OH	0.22(0.18,0.29)	0.19(0.15,0.24)	1.47 ± 0.92	1.06 ± 0.34	0.032
	Isovaleric academia, 2-Methylbutyryl-CoA dehydrogenase deficiency, Isobutyryl-CoA dehydrogenase deficiency	13	C5	0.11(0.08,0.14)	0.10(0.08,0.13)	1.35 ± 0.88	1.21 ± 0.92	0.603
Amino acid metabolic disease	Hyperphenylalaninemia	31	Phe	68.00(52.88,83.51)	52.05(46.34,59.08)	1.44 ± 0.59	1.05 ± 0.26	0.001
	Homocystinuria, Hypermethioninemia	10	Met	26.34(18.64,46.73)	21.21(17.15,25.98)	1.45 ± 0.70	1.04 ± 0.34	0.097
	Argininosuccinic aciduria, Citrullinemia Type I, Citrin deficiency	19	Cit	15.05(11.71,25.14)	12.51(10.32,15.56)	1.77 ± 1.55	1.10 ± 0.57	0.077
Fatty acid β oxidation disorder	Short-chain acyl-CoA dehydrogenase deficiency	16	C4	0.31(0.25,0.45)	0.21(0.18,0.25)	1.8 ± 0.98	1.05 ± 0.32	0.008
	Primary carnitine deficiency	34	C0	14.65(8.64,18.99)	21.87(16.65,26.28)	0.69 ± 0.31	1.07 ± 0.42	< 0.001

Note: Diseases with low-frequency carriers were not included in the analysis. Data of carriers and non-carriers were shown by median and inter quartile range, the concentration unit was µmol/L

We compared the occurrence of carriers among false-positive and negative newborns. False-positive results were defined as newborns with abnormal traditional biochemical results (the NBS result was considerably higher or lower than the cutoff value) and accepted for further assessment, such as clinical manifestations, individualised assistant examinations, and NGS panel tests but eventually diagnosed as healthy. The results showed that there was a higher occurrence of carriers among newborns who received false-positive results for amino acid metabolic diseases compared to those who received negative results (15.52% vs. 6.71%, *p* = 0.030) (Table 4). Similarly, there was a higher occurrence of carriers among newborns who received false-positive results for fatty acid β oxidation disorder compared to those who received negative results (28.30% vs. 7.29%, *p* < 0.001). Although not statistically significant, a similar trend was observed in organic acid metabolic diseases (*p* = 0.096).

**Table 4:** Comparation of the occurrence of carriers among false-positive and negative newborns

	Case	Carriers	Non-carriers	*P*
Congenital hypothyroidism				
false-positive	2	0	2	—
negative	686	58	628	
Organic acid metabolic disease				
false-positive	99	12 (12.12%)	87(87.87%)	0.096
negative	686	50 (7.29%)	636(92.71%)	
Amino acid metabolic disease				
false-positive	58	9 (15.52%)	49(84.48%)	0.030
negative	686	46 (6.71%)	640(93.29%)	
Fatty acid β oxidation disorder				
false-positive	53	15 (28.30%)	38(71.70%)	< 0.001
negative	686	50 (7.29%)	636(92.71%)	

Note: Carriers (or non-carriers) in this table refer to the number of carriers (or non-carriers) in false positive and negative newborns, which is different from the carrier group and non-carrier group in Table 3.

## Discussion

NBS is important for early disease detection, diagnosis, and intervention; however, current methods have high false-positive rates. In this study, we investigated the correlation between carried gene variants and high false-positive rates in newborns. Genomic sequencing showed that a large number of newborns in the study cohort were gene-variant carriers. Among the top ten common variants, *DUOX2*, *SLC22A5*, *PAH*, and *ACADS* were associated with CH, primary carnitine deficiency, hyperphenylalaninemia, and short-chain acyl-CoA dehydrogenase deficiency, respectively. The high carrying rates of these variants are consistent with the relatively high incidence of related IMDs in our previous multicentre study [6]. Of all the diseases with variants detected, organic acid metabolic, fatty acid β oxidation, CH, and amino acid metabolic diseases had relatively high variant rates, and their total variant rate was approximately 50%. All of these diseases are neonatal IMDs; therefore the carrying rate of variants related to IMDs was high among the newborns. Wang et al. [8] designed a newborn genetic-screening panel to detect pathogenic variants and found that 41.4% (29/70) of suspected positive newborns carried pathogenic gene variants found in traditional newborn biochemical screening, and 26% (13/50) of negative newborns carried pathogenic gene variants. The variant carrying rate in this study was higher (65.7%), which may be due to our data containing pathogenic variants as well as likely pathogenic and variants of uncertain significance. In addition, different NeoSeq pro panels contain different variation sites, which may explain why our variant-carrying rate differs from that of other studies.

Wang et al. [8] also revealed that carrying pathogenic gene variants may lead to an increase in the related biochemical indicators of the disease; however, no detailed analysis was performed owing to the small sample size. We analysed the relationships between the variants and the levels of biochemical indicators in newborns. We found that the biochemical indicators of most carriers were higher or lower than those of healthy newborns. Of the variants with relatively high frequencies, the biochemical indicators of carriers with organic acid metabolic, amino acid metabolic, and fatty acid β oxidation disorders were significantly increased or decreased compared to those of non-carriers, especially methylmalonic acidemia/propionate acidemia, short-chain acyl-CoA dehydrogenase deficiency, and primary carnitine deficiency. Hence, we believe that the carried variants lead to abnormalities in biochemical indicators. However, not all carriers had obvious biochemical abnormalities, and some smaller metabolic changes may not be clinically relevant or may be compensated by other pathways. For instance, despite that 56% of carriers exhibited elevated TSH levels compared to normal newborns, only two cases of false positive for CH were identified in our study population, both of which were not associated with any relevant genetic variants.

All newborns with a positive result need to be re-examined for diagnosis and identification of true and false positives. This process is time-consuming and is not conducive to the early diagnosis and treatment of diseases. It also increases parents’ anxiety and has persistent negative effects [9]. False-positive flagging is inherently dependent on the cutoff values for an NBS test, which are set independently by each laboratory (nationally or regionally). This is a methodological explanation for the high rate of false positives in NBS. False positivity can also be caused by other individual factors, such as preterm birth, low birth weight [10], jaundice [11], drug usage [12], maternal influence [13] and the quality of blood filter paper [14]. In this study, we found a higher occurrence of carriers among newborns who received false-positive results for fatty acid β oxidation disorder compared to those who received negative results, followed by amino acid and organic acid metabolic diseases. These findings are particularly important for explaining the high false-positive rate observed in fatty acid β oxidation disorders, which was previously thought to be due to the instability of acylcarnitine in blood filter paper or maternal effects [13, 15]. Our results indicate that carrying gene variants is an important cause of false positives in newborns, which can help interpret flagged NBS when confirmatory testing is pending, alleviate stress for parents during that period, and even eliminate persistent negative effects.

Similar to our findings, Parsons et al. [16] found that in NBS for cystic fibrosis (CF), the population with abnormal immunoreactive trypsinogen levels was enriched with carriers. Parents whose babies were identified as carriers were in favour of NBS and were no longer concerned about the health of their babies. Another study on CF showed that if children were NBS carriers or underwent further testing related to false-positive results, parents experienced the lasting effects of stress if they were not provided with timely and adequate information [17]. Once the parents understood the carrier results, they did not appear to experience lasting distress or anxiety. The challenge in the future is to explain carrier results and communicate with parents to ensure that they have sufficient information during the screening process.

One limitation of this study is that our NeoSeq Pro panel only contains 4,000 variations in 154 pathogenic genes. We realise that if more unscreened genes were included in the NeoSeq Pro panel, more newborns could be identified as carriers of variations in the unscreened genes. However, it is unknown whether these unscreened genes affect biochemical markers in newborns. The NeoSeq Pro panel chosen covered many variant sites related to IMDs, which were designed according to the literature and characteristics of common pathogenic genes in the Chinese population [6, 18]; therefore, we believe that our study is still significant.

## Conclusions

Our study illustrated that gene variant carriers related to IMDs comprise a large number of newborns. We analysed and confirmed that the carried variants could lead to abnormalities in biochemical indicators in newborns. We found that carriers are an important explanation for false-positive results in newborns with traditional biochemical screening. Our study provides information that can be used by clinicians to interpret false-positive neonatal disease screening results. Owing to the variety of diseases involving variant carrying, some diseases involving a small number of cases cannot be analysed for biochemical indicators; therefore, the impact of such genes on relevant biochemical indicators is unknown.

## Methods

### Study population

This retrospective study was conducted at the Department of Medical Genetics, Changzhou Maternal and Child Health Care Hospital (Changzhou, China). The study included 962 newborns who underwent traditional newborn biochemical screening from 2017 to 2022, including 222 cases with false-positive results and 740 cases with negative results. None of the newborns were identified as having metabolic diseases based on the original biochemical screening. This study was approved by the Institutional Ethics Committee of Changzhou Maternal and Child Health Care Hospital and followed the tenets of the Declaration of Helsinki. Written informed consent was obtained from all the study subjects.

### Traditional newborn biochemical screening

Four blood stains were collected on filter paper (Schleicher & Schuell 903) from the heel of newborns after six full lactation periods 72–96 h after delivery. The blood filter papers were allowed to air-dry in a horizontal position for at least 3 h and refrigerated at 2–8℃ for NBS and genomic sequencing. TSH levels were detected using a time-resolved fluoroimmunoassay with an AutoDELFIA Neonatal hTSH Kit (PerkinElmer, Shelton, CT, USA) [19]. The levels of 11 amino acids and 31 carnitines were evaluated by MS/MS using a Neobase TM Non-derivatised MSMS Kit (PerkinElmer) [6].

### Newborn genomic sequencing

Genomic sequencing was performed using the NeoSeq Pro panel (Hangzhou Biosan Clinical Laboratory Co., Ltd., Hangzhou, China), similar to our previously published study [7]. This panel can detect 154 pathogenic genes of 86 diseases with more than 17 categories. Briefly, genomic DNA was extracted from preserved blood stains of 962 newborns using a Nucleic Acid Automatic Extraction System (Bioer Technology, Hangzhou, China). DNA fragments were subjected to terminal repair, splicing, and PCR amplification to construct a pre-library, and liquid-phase hybridisation with specific capture probes for the target genome region was performed, customised on the Illumina Platform. DNBSEQ-T7 (MGI Tech, Shenzhen, China) was used for the high-throughput sequencing. Raw image files were processed using BCL in FASTQ (T7) to generate raw data. High-quality sequencing reads were aligned to the human reference genome from the National Center for Biotechnology Information human reference genome (hg19/GRCh37). The frequency of the variant sites in the normal population was determined using the Genome Aggregation Database (https://gnomad.broadinstitute.org/). Variant pathogenicity was determined using Online Mendelian Inheritance in Man (http://www.omim.org), ClinVar (http://www.ncbi.nlm.nih.gov/clinvar), and the Human Gene Variant Database (http://www.hgmd.org). All the target variants were subjected to biological effect analysis. Sanger sequencing was used to verify positive mutant sites.

### Statistical analyses

All analyses were performed using R software (version 3.4.3; http://www.R-project.org). For quantitative variables, normally distributed data were analysed using a two-sample t-test, and non-normally distributed data were analysed using the Mann–Whitney test. Categorical variables were compared using the chi-squared or Fisher’s exact tests. Two-tailed *p*-values < 0.05 were considered statistically significant.

### Electronic supplementary material

Below is the link to the electronic supplementary material.

**Supplementary Table 1:** Classification of diseases associated with 632 gene carriers

## Data Availability

The data that support the findings of this study are not openly available due to reasons of sensitivity and are available from the corresponding author upon reasonable request. Data are located in controlled access data storage at Changzhou Maternal and Child Health Care Hospital.

## Abbreviations

C0: free carnitine
C2: acetylcarnitine
C3: propionylcarnitine
C4: butyrylcarnitine
C4DC: methylmalonylcarnitine
C5OH: 3-hydroxyisovalerylcarnitine
CF: cystic fibrosis
CH: congenital hypothyroidism
Cit: citrullinaemia
IMD: inherited metabolic disease
Met: methionine
MS/MS: tandem mass spectrometry
NBS: newborn screening
NGS: next-generation sequencing
Phe: phenylalanine
TSH: thyroid-stimulating hormone
