# Supplementary material for: Abnormal biochemical indicators of neonatal inherited metabolic disease in carriers
Source: Orphanet J Rare Dis. 2024 Apr 4;19:145. doi: 10.1186/s13023-024-03138-5 (PMC10996179; doi:10.1186/s13023-024-03138-5)
Supplement: Supplementary file 1 — Supplementary Table 1: Classification of diseases associated with 632 gene carriers [file 13023_2024_3138_MOESM1_ESM.docx]

**Supplementary Table 1** Classification of diseases associated with 632 gene carriers

|  | Related disease types of carriers | NO. of case(percentage) |
| --- | --- | --- |
| 1 | Cholestasis | 303 (47.94) |
| 2 | Deafness | 162 (25.63) |
| 3 | Organic acid metabolic diseases | 104 (16.46) |
| 4 | Fatty acid β oxidation disorders | 84 (13.29) |
| 5 | Endocrine diseases | 74 (11.71) |
| 6 | Amino acid metabolic diseases | 70 (11.08) |
| 7 | lysosomal storage diseases | 44 (6.96) |
| 8 | Other inherited metabolic diseases | 42 (6.65) |
| 9 | Hematological system disorders | 32 (5.06) |
| 10 | Carbohydrate metabolism diseases | 26 (4.11) |
| 11 | Neuromuscular diseases | 5 (0.79) |
| 12 | Skeletal system disorders | 4 (0.63) |
| 13 | Immune system-related diseases | 4 (0.63) |
| 14 | Epilepsy | 1 (0.16) |
